# A comparative analysis of gene expression induced by the embryo in the caprine endometrium

**DOI:** 10.1002/vms3.221

**Published:** 2019-11-28

**Authors:** Xiaorui Liu, Lei Zhang, Jincheng Han, Lichun Yang, Jiuzeng Cui, Sicheng Che, Binyun Cao, Yuxuan Song

**Affiliations:** ^1^ College of Animal Science and Technology Northwest A&F University Yangling Shaanxi China

**Keywords:** Dairy goats, Differential expressed genes (DEGs), Endocytosis, Endometrium

## Abstract

Transcriptomics is an established powerful tool to identify potential mRNAs and ncRNAs (non‐coding RNAs) for endometrial receptivity. In this study, the goat endometrium at estrus day 5 (ED5) and estrus day 15 (ED15) were selected to systematically analyse the differential expressed genes (DEGs) what were induced by the embryo. There were 1,847 genes which were significantly differential expressed in endometrium induced by the embryo at ED5, and 1,346 at ED15 (*p*‐value < .05). Secreted phosphoprotein 1 (*SPP*) was the responsive genes for embryo in the goat endometrium during estrus cycle, neurotensis (*NTS*) and pleiotrophin (*PTN*) were the responsive genes for embryo in the goat endometrium at ED5, Testin (*TES*) and Phosphate and Tension Homology Deleted on Chromsome ten (*PTEN*) at ED15. Furthermore, Gene Ontology (GO) and Kyoto Encyclopedia of Genes (KEGG) analysis revealed cytoplasm and Endocytosis were indispensable for the endometrium development in dairy goat. In a word, this resulting view of the transcriptome greatly uncovered the global trends in mRNAs expression induced by the embryo in the endometrium of dairy goats.

## Graphical abstract

In this study, the goat endometrium at estrus day 5 and estrus day 15 were selected to systematically analyse the DEGs induced by the embryo in the endometrium of dairy goats, and there were 1,847 genes that were differential expressed at ED5, and 1,346 at ED15. In a word, this resulting view of the transcriptome greatly uncovered the global trends in mRNAs expression induced by the embryo in the endometrium of dairy goats.

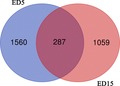

## 1 INTRODUCTION

Despite advances in our understanding of fertility, implantation failure remains a significant problem for both spontaneous and assisted pregnancies (Cha, Sun, & Dey, 2012). Implantation occurs when a free‐floating mature blastocyst attaches to the endometrium, invades the stroma and establishes the placenta. For this process to be successful, the endometrium must be in a receptive state (Teh, Mcbain, & Rogers, 2016), what was the result of the synchronized and integrated interaction among multiple factors and genes (Cha, Vilella, Dey, & Simon, 2013; Zhang et al., 2013).

Transcriptomics is a powerful tool to identify potential molecular biomarkers for endometrial receptivity, thereby improving forecasts of pregnancy outcome during in vitro fertilization treatment (Wang & Yu, 2018). Transcriptome analyses of distinct physiological stages were crucial for understanding the gene regulatory network, including the endometrium development in mammals (Wang et al., 2016; Zhang et al., 2015, 2017). Moreover, studies on the endometrial transcriptomics have been reported using RNA‐Seq in humans (Hu et al., 2014b), pigs (Lin et al., 2015; Samborski, Graf, Krebs, Kessler, & Bauersachs, 2013; Samborski, Graf, Krebs, Kessler, Reichenbach, et al., 2013; Wang et al., 2016), cattles (Mamo, Mehta, Forde, Mcgettigan, & Lonergan, 2001; Palma‐Vera, Sharbati, & Einspanier, 2015), and goats (Zhang et al., 2015, 2017).

In our previous studies, the goat endometrium at estrus day 5 (ED5) and estrus day 15 (ED15) were selected to systematically analyse the transcriptome of endometrium With embryo using strand‐specific Ribo‐Zero RNA‐Seq (Zhang et al., 2017). The most important and studied factor in the implantation is the embryo‐itself, however, there is no studies on the comparative analysis of mRNAs induced by the embryo in the endometrium of dairy goats. Then, we constructed a comprehensive analysis of the endometrial transcriptional profiles at the global level to compare the expressed mRNAs at ED5 and ED15, and further explore the differential expressed genes (DEGs). What's more, the Gene Ontology (GO) and Kyoto Encyclopedia of Genes (KEGG) were also analysed in the present study. Therefore, the results of the present study provided essential transcriptome to enhance the knowledge of mRNAs induced by the embryo in the endometrium of dairy goats.

## 2 MATERIALS AND METHODS

All animals in this study were maintained according to the No. 5 proclamation of the Ministry of Agriculture, P. R. China, and the animal protocols were approved by the Review Committee for the Use of Animal Subjects of Northwest A&F University.

### 2.1 Study design and sample collection

A total of 12 healthy, 24‐month‐old multiparous dairy goats (Xinong Saanen) were induced to oestrous synchronization for this study. The protocols were applied to goats as follows: each goat was injected with 0.2 mg prostaglandin F2α (Ningbo Pharmaceutical Co., Ltd., China). On the same day (day 1), goats were administered intravaginal sponges containing 60 mg medroxyprogesterone acetate MPA). On day 10, each goat was twice injected with 20 IU of follicle‐stimulating hormone (FSH) (Ningbo Pharmaceutical Co., Ltd.) (at 8.00 and 19.00 hr). On the day of sponge removal (day 11), each goat received an intramuscular injection of 0.1 mg prostaglandin F2α and 200 IU pregnant mare's serum gonadotropin (PMSG) (Ningbo Pharmaceutical Co., Ltd.). The experimental goats were observed three times daily to ascertain the estrous sign, and mated naturally twice in estrus (Lei et al., 2017; Liu et al., 2019; Zhang et al., 2015). The 12 experimental goats were observed three times daily to ascertained estrous sign, and the first day of estrus was considered to be day 0 (ED0). Six goats mated naturally twice, and the other six unmated. The goats were euthanized following intravenous injection of a barbiturate (30 mg/kg) at day 5 (ED5, *n* = 6, 3 mated and 3 unmated) and day 15 (ED15, *n* = 6, 3 mated and 3 unmated), and the endometrium samples were obtained from the anterior wall of the uterine cavity. All samples were washed briefly with PBS (Phosphate Buffered Saline) and then immediately frozen in liquid nitrogen.

### 2.2 RNA‐seq date

Total RNA was extracted from endometrium using TRIzol reagent (Invitrogen, CA, USA), and the DNA contamination was evaluated using DNase (TaKaRa, Dalian, China) according to the manufacturer's instructions. The total RNA quantity and purity were analysed using the Bioanalyzer 2100 (Agilent, CA, USA) and RNA 6000 Nano LabChip Kit (Agilent, CA, USA) with RIN number > 7.0. Library construction, sequencing, reads mapping were showed in our previous studies(Lei et al., 2017; Liu et al., 2019).

### 2.3 Differential expression of mRNAs in goat endometrium

Only comparisons with “q‐values” less than 0.01 and statuses indicated as “OK” in the Cuffdiff output were regarded as showing differential expression. The fold‐changes (log2 (ED15/ED5)) and corresponding significance threshold of the *p*‐value were estimated according to the normalized mRNA expression levels. Based on the expression levels, the significant DECs between the ED15 and ED5 were identified using “*p* < .05” as the threshold.

### 2.4 GO enrichment and KEGG pathway analysis

The hypergeometric test was applied to map all differentially expressed genes to terms in the Gene ontology (GO) database (ftp://ftp.ncbi.nih.gov/gene/DATA/gene2go.gz) (Consortium, 2004), which was an international standard gene functional classification system (Ashburner et al., 2000). The corrected *p*‐value < .05 was used as the threshold to find significantly enriched GO terms in the input list of DEGs compared to their genomic background.

KEGG was the major public pathway‐related database that helped to further understand the biological functions of genes with high level functions and the utilities of the biological system from large‐scale molecular datasets (http://www.genome.jp/kegg/) (Guo et al., 2014). KEGG enrichment analysis identified significantly enriched metabolic pathways or signal transduction pathways (He & Liu, 2013) using the corrected P‐value < 0.05 as a threshold to find significantly enriched KEGG terms in the input list of DEGs compared to The calculating formula of the corrected P‐value:

$$P=1-\sum_{i=0}^{m-1}\frac{\left(\frac{M}{i}\right)\left(\frac{N-M}{n-i}\right)}{\frac{N}{n}}$$

The *N* represented the number of GO/KEGG annotated genes in genome, *n* represented the number of differentially expressed genes in *N*. *M* represented the number of particular GO/KEGG annotated genes in genome, m represented the number of particular GO/KEGG annotated genes expressed differentially in *M* (Ji et al., 2012).

## 3 RESULT

### 3.1 Differential expression of mRNAs (DEGs) induced by the embryo in the endometrium of dairy goats

At ED5, 1,847 genes were differential expressed in endometrium with and without embryo, including *SPP1, IL18, WNT16, WNT2B, WNT2, HOXA6, LIFR, NTS, PTGS2, CXCL14, S100A2, BCL2L15, SUSD2, PTN* (Data S1). At ED15, 1,346 genes were differential expressed in endometrium between with and without embryo, including *SPP1, IL1R2, IL18R1, IL17RC, ILDR2, IL10RB, S100A10, S100A4, S100A14, S100G, S100A2, IGF‐I, GPX8, VEGFC, MAP3K7* *, PIK3CA, IGFBP7, PTEN* (Data S2).

### 3.2 The common DEGs at ED5 and ED15 in the endometrium

**Figure 1:**
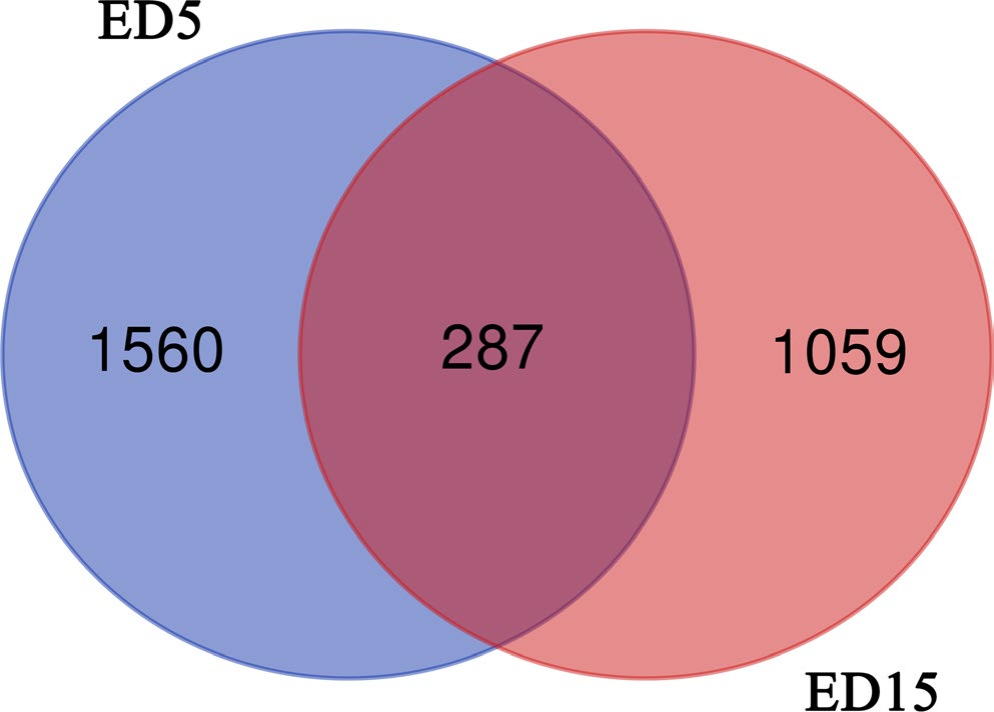
Venn diagrams of differential expressed genes (DEGs) in goat endometrium with or without embryo at ED5 and ED15

In total, 2,906 DEGs were found, and 287 of these were co‐expressed at both stages, 1,560 DEGs only were found at ED5, 1,059 DEGs were found at ED15 (Figure 1, Data S3). And then we pay more attention to the 1,059 DEGs at ED15, because these genes may be the responsive genes for the embryo. In other words, the expression levels of these 1,059 genes were regulated by embryo directly or indirectly in endometrium. Among these genes, *TES* and *PTEN* got our attention, because our previous studies showed that they play important roles in the endometrial cells during the development of endometrium in dairy goats (Zhang et al., 2017).

### 3.3 GO enrichment analysis

The DEGs were analysed by running queries for each DEG against the GO database, which provided information related to three ontologies: molecular function, cellular component and biological process.

**Figure 2:**
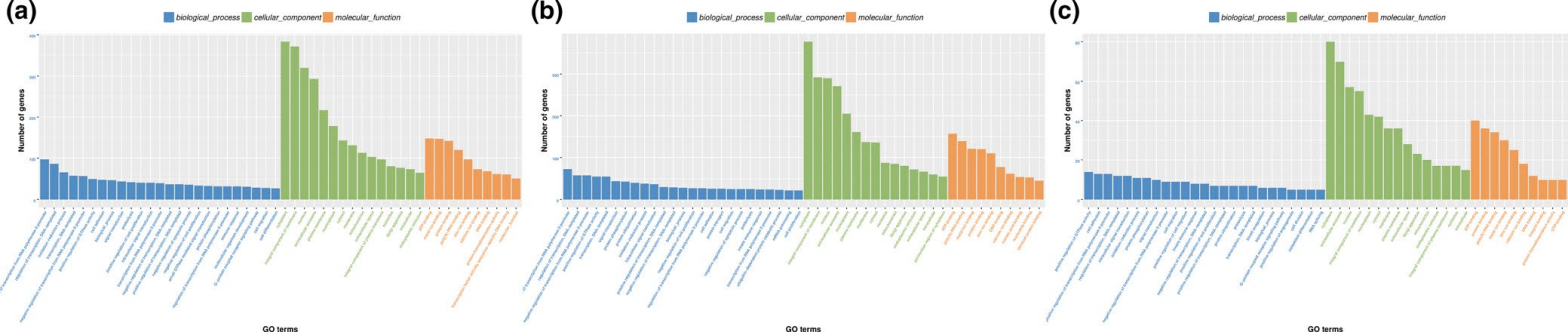
The GO enrichment analysis of the DEGs. (a) the GO enrichments of the DEGs at ED5; (b) the GO enrichments of the DEGs at ED5; (c) the GO enrichments of the common DEGs at both ED5 and ED15. Note: the x‐axis indicated the specific category of GO, the y‐axis indicated the percent of DEGs

At ED5, GO enrichments of the DEGs of ED5 were categorized into 663 functional groups that met the criteria of *p*‐values < .05 (Data S4), and the distribution diagram was shown in Figure 2a. Of the 148 terms that were significantly enriched in molecular functions, the significantly enriched GO term with the most DEGs was GO:0015440 (peptide‐transporting ATPase activity) what was annotated with 148 DEGs, followed by GO:0046872 (metal ion binding) with 147 DEGs. In the cellular compartment GO category, 83 terms were significantly enriched. The significantly enriched GO term with the most DEGs was GO:0005737 (cytoplasm) what was annotated with 383 DEGs, followed by GO:0005634 (nucleus) with 320 DEGs. In the biological processes, 432 GO terms were significantly enriched and associated with various processes, such as GO:0045944 (positive regulation of transcription from RNA polymerase II promoter) with 97 DEGs annotated, GO:0043547 (positive regulation of GTPase activity) with 50 DEGs and GO:0007155 (cell adhesion) with 48 DEGs.

At ED15, GO enrichments of the DEGs of ED15 were categorized into 578 functional groups that met the criteria of *p*‐values < .05 (Data S5), and the scatter diagram was shown in Figure 2b. Of the 125 terms that were significantly enriched in molecular functions, the significantly enriched GO term with the most DEGs was GO:0005524 (ATP binding) what was annotated with 157 DEGs, followed by GO:0044822 (poly(A) RNA binding) with 139 DEGs. In the cellular compartment GO category, 115 terms were significantly enriched. The significantly enriched GO term with the most DEGs was GO:0005737 (cytoplasm) what was annotated with 378 DEGs, followed by GO:0005634 (nucleus) with 290 DEGs. In the biological processes, 338 GO terms were significantly enriched and associated with various processes, such as GO:0000122 (negative regulation of transcription from RNA polymerase II promoter) with 58 DEGs annotated, GO:0043547 (positive regulation of GTPase activity) with 55 DEGs and GO:0006468 (protein phosphorylation) with 43 DEGs. These results suggested that some DEGs increased or decreased in response to embryo.

What's more, GO enrichments of the common DEGs were categorized into 492 functional groups that met the criteria of *p*‐values < .05 (Data S6), and the scatter diagram was shown in Figure 2c. Of the 106 terms that were significantly enriched in molecular functions, the significantly enriched GO term with the most DEGs was GO:0005524 (ATP binding) what was annotated with 40 DEGs, followed by GO:0005515 (protein binding) with 36 DEGs. In the cellular compartment GO category, 82 terms were significantly enriched. The significantly enriched GO term with the most DEGs was GO:0005737 (cytoplasm) what was annotated with 80 DEGs, followed by GO:0070062 (extracellular exosome) with 70 DEGs. In the biological processes, 304 GO terms were significantly enriched and associated with various processes, such as GO:0043547 (positive regulation of GTPase activity) with 15 DEGs annotated.

The GO analysis showed that GO:0005737 (cytoplasm) was the significantly enriched GO term with the most DEGs at both ED5, ED15 and common group, suggesting that the annotated genes might play important roles in the goat developmental endometrium with embryo during estrus cycle, such as *TES* and *PTEN* at ED15.

### 3.4 KEGG pathway analysis

**Figure 3:**
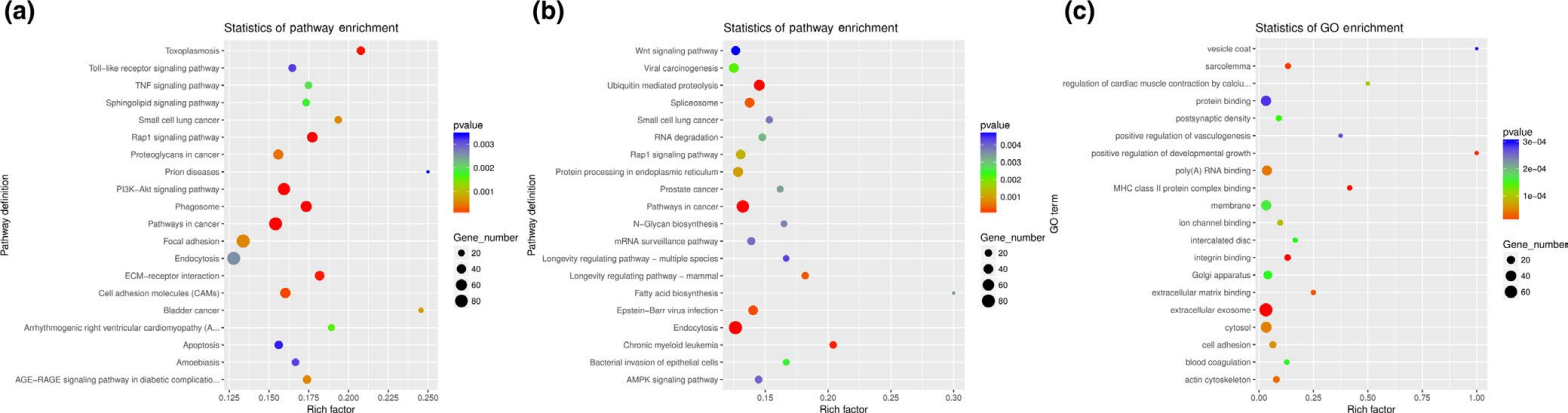
Scatter plot of the KEGG pathway enrichment analysis of the DEG. (a) the KEGG analysis of the DEGs at ED5; (b) the KEGG analysis of the DEGs at ED5; (c) the KEGG analysis of the common DEGs at both ED5 and ED15. Note: The x‐axis indicates the number of ctDElncRs assigned to a specific pathway, the y‐axis indicates the pathway. The pathway enrichment statistics were performed by Fisher's exact test, and with a corrected *p* < .05 were considered the significant pathways

Various genes cooperated with each other to exercise their biological functions. Accordingly, the KEGG analysis helped us further understand the biological functions of DEGs (Kanehisa et al., 2014), and the scattergram for ED5 were shown in Figure 3a. The DEGs were significantly enriched in 64 KEGG pathways meeting the criteria of *p*‐values < .05 (Data S7), suggesting that these pathways might play important roles in the development of endometrial receptivity. The KEGG pathways with the highest levels of significance were the map04510 (Focal adhesion) with 88 DEGs, followed by map04144 (Endocytosis) with 84 DEGs. At ED15 (Figure 3b), the DEGs were significantly enriched in 60 KEGG pathways meeting the criteria of *p*‐values < .05 (Data S8), suggesting that these pathways might play important roles in the development of endometrial receptivity. The KEGG pathways with the highest levels of significance were the map04144 (Endocytosis) with 84 DEGs, followed by map05200 (Pathways in cancer) with 84 DEGs. As to the common DEGs (Figure 3c), significantly enriched in 37 KEGG pathways meeting the criteria of *p*‐values < .05 (Data S9), suggesting that these pathways might play important roles in the development of endometrial receptivity. The KEGG pathways with the highest levels of significance were the map04810 (Regulation of actin cytoskeleton) with 22 DEGs, followed by map04144 (Endocytosis) with 21 DEGs.

The KEGG analysis showed that map04144 (Endocytosis) was significantly enriched at both ED5, ED15 and common group suggesting that Endocytosis might participate in the goat developmental endometrium whether with embryo or not during estrus cycle.

## 4 DISCUSSIONS

Many global transcriptomic studies of human endometrium have been published in the past years, providing considerable information on the likely pattern of expression in receptive endometrium. Altmäe et al. (2014) summarized a comprehensive review of omics studies in human endometrium and discovered that the number of genes identified in more than one study as potential biomarkers in endometrial physiology and pathophysiology has remained small, while any given study yields numerous candidate genes to explore.

In 2013, the domestic goat genome was generated using Whole Genome Mapping technology (Dong et al., 2013), which endowed its high quality of genome assembly and annotation. Currently, Ribo‐Zero RNA‐Seq is a competitive method to investigate the gene expression, particularly for incompletely annotated genomes (Derks et al., 2015; Sun et al., 2015). Our previous study reported the systematic identification and characterization of the developmental transcriptome landscape of the goat endometrium without embryo during estrus cycle using the Ribo‐Zero RNA‐Seq technology (Zhang et al., 2017). What was of significantly higher depth, yielding 18 Gb of sequence for every sample, which was approximately seven times the size of the goat genome (2.66 Gb).

Some studies have reported modifications in gene expression profiles associated with transition of the human endometrium from a pre‐receptive to a receptive stage (Cuevas et al., 2016; Díaz‐Gimeno et al., 2011; Hu et al., 2014a; Sigurgeirsson et al., 2017). However, only two genes were common to six of the seven largest studies, secreted phosphoprotein 1 (SPP1; previously known as osteopontin) (Wang & Yu, 2018). SPP1 is a glycoprotein involved in cellular adhesion and migration. It is the only factor identified that is common to most reported endometrial receptivity gene sets (Ruiz‐Alonso et al., 2013), and it is reported to be an essential mediator of implantation and receptivity in humans (Gibson, Simitsidellis, Cousins, Critchley, & Saunders, 2016), what was confirmed in goats in this study.

Previously, much attention was devoted to the function of neurotensis (*NTS*) in cancer (Kontovounisios, Qiu, Rasheed, Darzi, & Tekkis, 2017), or as an important regulator of the fat absorption and obesity (Jing et al., 2016). NTS was detected in bovine endometrium, and the expression levels showed difference between the breeding and non‐breeding seasons (Sakumoto et al., 2015). In previous study, NTS could promote EECs proliferation and inhibit cell apoptosis in vitro, by increasing BCL‐2 and decreasing BAX. What's more, NTS could increase some biochemical markers of receptive endometrium, such as LIF, COX2 and HOXA10 in EECs in vitro. However, it should be noted that there was no significant changes on the protein levels of LIF, COX2 and HOXA10 when the NTS was knockdown partly by specific siRNA in EECs. All these results suggested that NTS was helpful to the formation of endometrial receptivity by increasing the protein levels of LIF, COX2 and HOXA10, but this function was not absolutely necessary in EECs in vitro.

PTN (also called HBGF8), an 18‐kDa heparin‐binding growth factor, shares 50% sequence homology with midkine (Blondet, Carpentier, Lafdil, & Courty, 2005; Deuel, Zhang, Yeh, Silossantiago, & Wang, 2002; Kadomatsu & Muramatsu, 2004; Pufe, Bartscher, Petersen, Tillmann, & Mentlein, 2003). It is a secreted cytokines (Li et al., 1990) that plays roles in diverse biology process, such as cell adhesion, migration, survival, growth and differentiation (Deuel et al., 2002; Kadomatsu & Muramatsu, 2004; T. Muramatsu, 2002). Further study showed abnormalities on reproduction and development were observed in the *PTN*‐KO mice (Muramatsu et al., 2006). What's more, *PTN* was stimulated by pregnancy, displayed a higher expression in the caruncular areas over the intercaruncular areas in bovine (Mansouriattia et al., 2009), and it increased in murine implantation sites during decidualization (Bany & Schultz, 2001). Considering the fact that *PTN* mainly expressed in the C areas of bovine endometrium, it may participate in the proliferation of stroma cells, and in the decidualization‐like process (Johnson et al., 2003) in dairy goats. What's more, the protein levels of *PTN* increased with the increase in *NTS* in EECs (Zhang et al., 2017). Considering the fact that *PTN* mainly expressed in the caruncular areas of bovine endometrium (Mansouriattia et al., 2009), it might participate in the proliferation of endometrial cells in dairy goats.

In this study, *NTS* was down‐regulated and *PTN* was up‐regulated genes what were induced by the embryo in the endometrium of dairy goats at ED5. And more in‐depth research was needed to study the functions of *NTS* in EECs of in dairy goats.

The changes in endometrial *PTEN* expression at both the mRNA and protein levels throughout the menstrual cycle were cyclic changes (Mutter, Lin, Kum, & Eng, 2000). Endometrial *PTEN* expression revealed temporal and spatial changes throughout the menstrual cycle, and during early pregnancy, E2 might down‐regulate *PTEN* activity by increasing its phosphorylation. However, P4 was likely to regulate the *PTEN* pool by decreasing its phosphorylation and increasing its protein level (Guzeloglukayisli et al., 2003). Thus, we hypothesized that *PTEN* expression was also regulated by E2 and P4 in goat endometrium cells, and this conjecture was verified in the previous study in which we observed a direct regulation of *PTEN* by E2 and P4 in the EECs and ESCs of dairy goats (Zhang et al., 2017).

*TES* is expressed in all normal human tissues (Sarti et al., 2005). It is localized in the cytoplasm as a component of focal adhesions and cell‐cell connections (Coutts, Mackenzie, Griffith, & Black, 2003; Griffith, Coutts, & Black, 2005). The protein encoded by the *TES* gene is a negative regulator of cell growth and may also function as a tumour suppressor (Steponaitis et al., 2016), as the loss of *TES* expression is frequently documented in various cancers (Z, 2014; Mcfarlane, 2001; Tatarelli, Linnenbach, Mimori, & Croce, 2000; Weeks, Ludgate, Lemée, & Morison, 2016; Zhong, Zhang, & Yin, 2015). In our previous study, it is observed that *TES* can promote EEC proliferation and inhibit cell apoptosis in vitro. *TES* could decrease BAX protein levels, and increase FAS in EECs in vitro, which is a critical factor for the progression of extrinsic apoptosis in human endometrum (Harada et al., 2004; Otsuki, 2001). Hence, we hypothesized that *TES* inhibited EECs apoptosis by decreasing the expression level of BCL‐2/BAX via the MAPK pathway. Thus, modulation of *TES* expression in EECs may emerge as a potential target in regulating the development of pre‐receptive endometrium in dairy goats.

In this study, *TES* and *PTEN* were up‐regulated genes induced by the embryo in the endometrium of dairy goats at ED15 but not at ED5, and more in‐depth research was needed to study the functions of NTS in EECs of in dairy goats.

During the implantation window, four endometrial cell types are identified in distinct proportions: microvilli‐rich cells, pinopode cells, ciliated cells and others without apical differentiation. Zhioua highlighted important differences between surface and glandular epitheliums (Zhioua et al., 2012). Using transmission electron microscopy (TEM) for ultrastructural analysis, Zhioua showed images of endocytosis in epithelial cells of the endometrium, suggesting each cell type and each cell structure as a very precise function in order to prepare the endometrium to be receptive.

During the process of endocytosis cells engulf extracellular fluid (ECF) into plasma membrane invaginations which are then pinched off to form intracellular membrane‐bounded vesicles, this is referred to as membrane mediated endocytosis (Quinn, Folkard, Detmar, & Casper, 2008). What's more, progesterone (P4) could control the endocytotic activity of the uterine epithelium and/or the movements of the endocytotic vesicles within the cells is supported by previous studies in the rat, as endocytosis is observed at Day 5 of pregnancy and during pseudopregnancy but not in cyclic animals (Vokaer & Leroy, 1962). Endocytosis occurred in pregnant and non‐pregnant cows but was especially marked when circulating progesterone concentrations were high, and there was no evidence that pinopod‐like functions could be attributed to large cytoplasmic protrusions from endometrial cells (Guillomot, Betteridge, Harvey, & Goff, 1986).

The GO and KEGG analysis showed that GO:0005737 (cytoplasm) and map04144 (Endocytosis) were significantly enriched with the most DEGs at both ED5, ED15 and common group, suggesting that cytoplasm and Endocytosis were indispensable for the goat developmental endometrium whether with embryo or not during estrus cycle.

## 5 CONCLUSIONS

In this study, the goat endometrium at estrus day 5 and estrus day 15 were selected to systematically analyse the DEGs induced by the embryo in the endometrium of dairy goats. There were 1,847 genes that were differential expressed at ED5, and 1,346 at ED15. *SPP1* was the responsive up‐regulated gene for embryo in the goat endometrium during estrus cycle, *NTS* was responsive down‐regulated and *PTN* was responsive up‐regulated genes for embryo in the goat endometrium at ED5, *TES* and *PTEN* were responsive up‐regulated genes at ED15. Further GO and KEGG analysis revealed that GO:0005737 (cytoplasm) and map04144 (Endocytosis) were indispensable for the endometrium development in goat. In a word, this resulting view of the transcriptome greatly uncovered the differential expressed genes induced by the embryo in the endometrium of dairy goats.

## 6 Implications

In this study, the goat endometrium at estrus day 5 and estrus day 15 were selected to systematically analyse the DEGs induced by the embryo in the endometrium of dairy goats. There were 1,847 genes that were differential expressed at ED5, and 1,346 at ED15. *SPP1* was the responsive genes for embryo in the goat endometrium during estrus cycle, *TES* and *PTEN* were the responsive genes for embryo in the goat endometrium at ED15. Further GO and KEGG analysis revealed that GO:0005737 (cytoplasm) and map04144 (Endocytosis) were indispensable for the endometrium development in goat. In a word, this resulting view of the transcriptome greatly uncovered the global trends in mRNAs expression induced by the embryo in the endometrium of dairy goats.

## CONFLICT OF INTEREST

The authors declare that they have no competing interests.

## ETHICAL STATEMENT

In this study, the Review Committee for the Use of Animal Subjects of Northwest A & F University approved the animal protocols. And all experimental dairy goats were maintained according to the No. 5 proclamation of the Ministry of Agriculture, P. R. China.

## Data Availability

The data that support the findings of this study are available from the corresponding author upon reasonable request.
